# Systemic Artery to Pulmonary Artery Shunt Mimicking Acute Pulmonary Embolism, Unmasked by a Multimodality Imaging Approach

**DOI:** 10.3390/tomography8010014

**Published:** 2022-01-07

**Authors:** Brieg Dissaux, Pierre-Yves Le Floch, Romain Le Pennec, Cécile Tromeur, Pierre-Yves Le Roux

**Affiliations:** 1 Radiology Department, University Hospital of Brest, 29609 Brest, France; lefloch.pierreyves29@gmail.com; 2 Nuclear Medicine Department, University Hospital of Brest, 29609 Brest, France; romain.lepennec@chu-brest.fr; 3 Pneumology Department, University Hospital of Brest, 29609 Brest, France; cecile.tromeur@chu-brest.fr

**Keywords:** Computerized Tomography (CT), imaging, diagnostic testing, embolism, vascular disease

## Abstract

In this report, we describe the functional imaging findings of systemic artery to pulmonary artery shunt in V/Q SPECT CT imaging. A 63-year-old man with small-cell lung cancer underwent CT pulmonary angiography (CTPA) for suspected acute pulmonary embolism (PE). The CTPA showed an isolated segmental filling defect in the right lower lobe, which was initially interpreted as positive for PE but was actually the consequence of a systemic artery to pulmonary artery shunt due to the recruitment of the bronchial arterial network by the adjacent tumor. A V/Q SPECT/CT scan was also performed, demonstrating a matched perfusion/ventilation defect in the right lower lobe.

## 1. Introduction

The diagnosis of acute pulmonary embolism (PE) on computed tomography pulmonary angiography relies on the identification of an endoluminal defect in the pulmonary arterial network. However, there are other causes of filling defects that can mimic PE. 

## 2. Case Presentation

A 63-year-old man was admitted to the emergency department with acute chest pain. This patient suffered from a small-cell lung cancer with pleural carcinomatosis and had been treated with Crizotinib for 30 months. The patient was apyretic and eupneic. Chest X-ray showed an alveolar condensation in the right lower lobe that was related to the known neoplasia. A cardiac cause was ruled out by an ECG and a troponin assay. The hypothesis of a pulmonary embolism (PE) was then suggested. The patient had an intermediate clinical pre-test probability of PE. A D-Dimer assay was positive at 880 µg/L.

**Figure 1:**
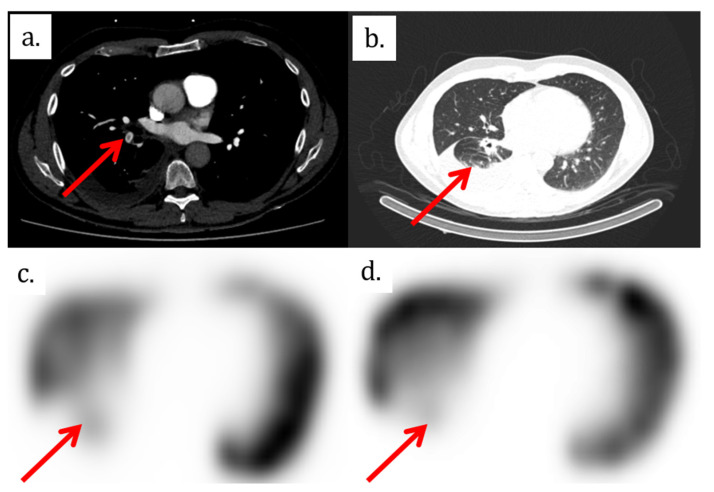
(**a**) Axial view of CTPA shows a segmental filling defect in a segmental artery of the right lower lobe. (**b**) Lung CT images show retractile neoplastic lesion of the right inferior lobe with a low right pleural effusion. (**c**) and (**d**) show perfusion and ventilation SPECT images, respectively, with a matched defect in the right lower lobe.

**Figure 2:**
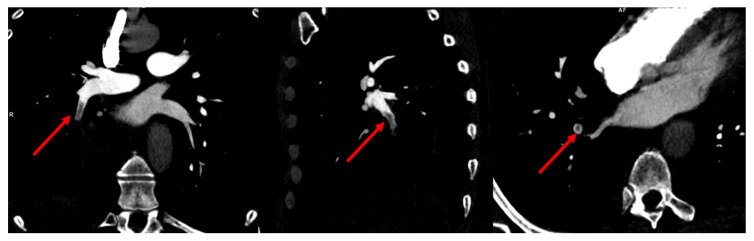
Multiplanar reformat views of CTPA showing a segmental filling defect in a segmental artery of the right lower lobe.

**Figure 3:**
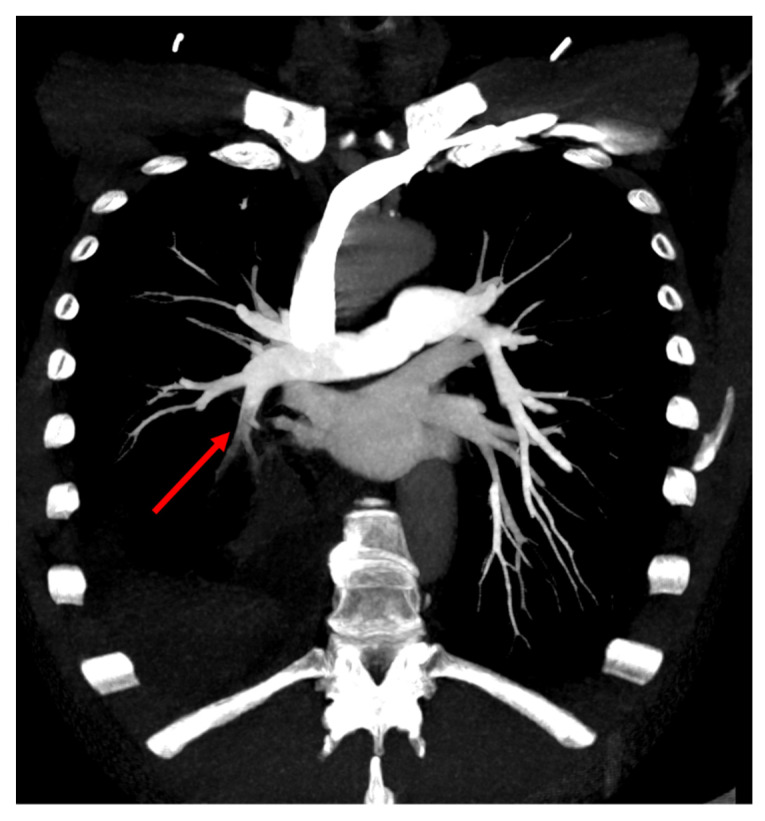
Maximum intensity projection reformat of CTPA showing a segmental filling defect in a segmental artery of the right lower lobe.

The patient therefore underwent a computed tomography pulmonary angiography (CTPA), which showed an isolated filling defect in the segmental artery of the posterior basal segment of the right inferior lobe (Figure 1, Figure 2 and Figure 3). CTPA also showed a known retractile neoplasic lesion of the right inferior lobe with a low right pleural effusion. The patient was hospitalized and treated with anticoagulation therapy (Tinzaparine). Then, the patient was enrolled in a trial to assess the diagnostic accuracy of iodine-map CTPA for the segment-based evaluation of lung perfusion in patients with acute PE [1]. As part of this trial, he underwent ventilation/perfusion single-photon-emission-computed tomography/computed tomography (V/Q SPECT/CT) 15 h after the CTPA. V/Q SPECT/CT did not show a perfusion mismatched defect, which excluded a PE diagnosis (Figure 1) [2,3]. V/Q SPECT images only showed a matched defect in the right lower lobe both in perfusion and ventilation (Figure 1).

**Figure 4:**
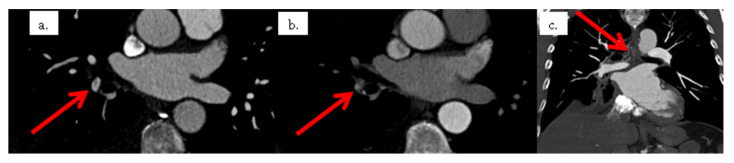
Second CT scan with two acquisition times. (**a**) shows axial early-acquisition CT with a segmental filling defect in a segmental artery of the right lower lobe. (**b**) shows axial late acquisition CT with, instead of a filling defect, the high enhancement of the same segmental pulmonary artery and of the bronchial arteries, while the other pulmonary arteries were not enhanced. (**c**) shows a coronal maximum intensity projection reformat of the late acquisition CT. It shows dilated systemic bronchial arteries feeding the right inferior lobar pulmonary neoplastic.

Due to this discrepancy, lower limb compression ultra-sonography was performed but did not show any proximal or distal deep vein thrombosis. CTPA images were reviewed by two radiologists. They confirmed the presence of a single filling defect in a segmental artery of the right lower lobe. However, the relatively low hypodensity of the endoluminal defect was not typical of PE. A new CTPA with two acquisition times was therefore performed 2 days later. The early acquisition, assessing the arterial pulmonary vessels, showed again a single filling defect in a segmental artery of the right lower lobe. A delayed acquisition assessing the aorta and its branches was therefore performed. Delayed images did not show the filling defect in the segmental artery of the right lower lobe. In contrast, there was high enhancement in the exact location of the filling defect on early acquisition. Furthermore, delayed images showed the opacification of the right lobar inferior vessels from dilated bronchial arteries from the aortic cross (Figure 4), confirming the diagnosis of a systemic to pulmonary artery shunt.

## 3. Discussion

The pulmonary vasculature is made up of two systems: the nourishing bronchial arterial network and the functional pulmonary arterial network that ensures hematosis. There are physiological connections between these two systems at a capillary and pre-capillary level. However, it is extremely rare to detect them radiologically [4,5]. The systemic artery to pulmonary artery shunts are usually diagnosed early in the lifetime when they are due to congenital heart or lung disease. In adults, the majority of the connections between the bronchial and pulmonary arterial networks are acquired. Transpleural systemic-pulmonary artery anastomoses can develop in patients with pleural adhesion, issuing from intercostal arteries, internal mammary arteries, inferior phrenic arteries, branches of the thyrocervical trunk, branches of the hepatic arteries, abdominal arteries, or other regional arteries [6]. However, most of the time, systemic to pulmonary artery anastomoses occur between the bronchial arteries and the peripheral branches of the pulmonary artery. In certain circumstances, such as chronic inflammatory disease or neoplasic lesions, the pulmonary artery network is defective and is not able to ensure vascularization on its own.

The bronchial arterial network is therefore recruited to make up for the lack. The bronchial arterial network is at high pressure and the pulmonary arterial network is at low pressure. The development of this high pressure vascularization is at risk of hemoptysis, but can also reopen pre-existing anastomoses at the pre- and post-capillary levels between the pulmonary arterial network and the bronchial arterial network. The re-opening of these anastomoses results in a shunt, which may be anterograde or retrograde, between the systemic arterial network at high pressure and the pulmonary arterial network at low pressure [7]. Depending on the circumstances and when the pulmonary parenchyma remains preserved, the shunt will be more frequently anterograde towards the pulmonary veins. Otherwise, the shunt will be rather retrograde, with drainage towards the pulmonary arteries.

The diagnosis of systemic artery to pulmonary artery shunt can be made by CT angiography (CTA) at a systemic arterial timing, allowing the opacification of the aortic cross and the bronchial arteries. The bronchial artery is the main source of systemic blood supply to the lungs. The normal bronchial artery is small, nearing 2 mm diameter, and arising from the aortic cross. In the case of a systemic artery to pulmonary artery shunt, CTA could demonstrate the dilatation of the bronchial artery and its branches and the underlying pathology (neoplastic lesion, chronic inflammatory disease). Surgery or transcatheter embolization may be required to treat massive bleeding [6].

CTPA is a well-established test for the diagnosis of PE [8]. The diagnosis of PE is based on the identification of an endoluminal defect in the pulmonary arterial network. Systemic artery to pulmonary artery shunt is a rare cause of filling defects in the pulmonary arteries on CTPA [7]. Other common causes of filling defects include motion artifacts, low contrast enhancement, or streak artifacts [4]. Pulmonary artery sarcomas have also been reported to mimic pulmonary embolism by showing the proximal filling defect [9].

CTPA, when used for the diagnosis of PE, is performed at the pulmonary artery time. A quality criterion of the CTPA in this indication is a low level of opacification of the systemic network and therefore of the aorta. However, in the rare context of a systemic arterial-pulmonary shunt, there may be a flow phenomenon within the pulmonary arterial network, which appears washed by non-opacified blood from the systemic bronchial arterial network originating from the aorta or its branches. For this reason, some authors have recommended slightly later acquisition with the satisfactory opacification of the thoracic aorta [7].

In our patient, the first CTPA showed a filling defect due to the shunt. The non-opaque blood from the systemic arterial network countercurrently washed the opaque pulmonary arterial blood with the iodinated contrast material. In the present case, this was more evident because there was no aortic enhancement and therefore no iodinated contrast in the systemic circulation. This shunt was due to the recruitment of the bronchial arterial network by the adjacent pulmonary neoplasic lesion.

At the second CTPA scan, a delayed acquisition was performed, allowing an opacification of the bronchial arterial network. This delayed acquisition made it possible to opacify the enlarged bronchial arterial network and countercurrently opacify the pulmonary arterial network in the right lower lobe, ruling out the presence of a clot in these arteries.

While the patient was deemed to be treated with long-term anticoagulant therapy, the hypothesis of a wrong diagnosis was raised by a negative V/Q SPECT/CT scan performed as part as a clinical trial. Interestingly, V/Q SPECT showed a matched defect in both perfusion and ventilation images in the right lower lobe. The principle underlying V/Q scintigraphy is straightforward. For perfusion images, macroaggregated albumin (MAA) particles are trapped on first-pass in the lung capillaries so that local concentration is related to the regional pulmonary blood flow at an arteriolar level [10]. In the present case, because of the reverse systemic arterial flow in the right lower lobe, MAA did not go through these arteries but towards other territories. However, while there was a perfusion defect, the diagnosis of PE was ruled out because of a matched ventilation defect in the right lower lobe. This may be explained by chronic bronchoconstriction resulting from local functional hypoperfusion secondary to the shunt, which did not allow correct hematosis. This clinical case illustrates that the mismatched V/Q defect is the key finding of acute PE in lung scintigraphy and that any matched defects could suggest another etiology.

## 4. Conclusions

This clinical case illustrates that the mismatched V/Q defect is the key finding of acute PE on lung scintigraphy and that any matched defects should suggest other etiology. Systemic artery to pulmonary artery shunt is a rare cause of filling defects in the pulmonary arteries on CTPA.

## Data Availability

No new data were created or analyzed in this study. Data sharing is not applicable to this article.
